# A novel *PIKFYVE* mutation in fleck corneal dystrophy

**Published:** 2011-10-25

**Authors:** Andreas Kotoulas, Haris Kokotas, Konstantinos Kopsidas, Konstantinos Droutsas, Maria Grigoriadou, Hasret Bajrami, Daniel F. Schorderet, Michael B. Petersen

**Affiliations:** 1 Department of Ophthalmology, General Hospital of Nafplio, Greece; 2 1st Department of Ophthalmology, Athens University Medical School, General Hospital “G. Gennimatas,” Greece; 3 Department of Genetics, Institute of Child Health, Athens, Greece; 4 Institute for Research in Ophthalmology, Sion, Switzerland; 5 Department of Ophthalmology, University of Lausanne, Lausanne, Switzerland; 6 Faculty of Life Sciences, Ecole Polytechnique Fédérale de Lausanne and University of Lausanne, Lausanne, Switzerland

## Abstract

**Purpose:**

To report the findings of the clinical and molecular evaluation in a Greek family with fleck corneal dystrophy (CFD).

**Methods:**

A 58-year-old woman was seen on routine ophthalmic examination and diagnosed as having CFD. All available family members were examined to evaluate the clinical findings and inheritance of the disease. Twenty members of the family in five generations underwent slit-lamp examination. Eleven were females and nine males, aged from two years to 85 years old. Blood samples were available from four patients with CFD and seven unaffected relatives, and the DNAs were subjected to molecular screening of the phosphoinositide kinase, five finger-containing (*PIKFYVE*) gene by direct sequencing or denaturing high performance liquid chromatography (DHPLC).

**Results:**

The clinical evaluation revealed six family members (five females and one male) with CFD. In two CFD patients early cataract formation was noticed. All patients affected with the corneal dystrophy were asymptomatic. The molecular analyses demonstrated the existence of a novel c.3060–3063delCCTT (p.P968Vfs23) mutation in *PIKFYVE* in all CFD patients tested but in none of the six unaffected family members. No molecular screening was performed in the seventh unaffected member as the causative mutation was clearly transmitted from his affected wife to his affected son.

**Conclusions:**

We report on the clinical and molecular findings of a five generation Greek family with CFD and we conclude that the novel c.3060–3063delCCTT (p.P968Vfs23) mutation in *PIKFYVE*, which segregated with the disease, was the causative mutation in this family.

## Introduction

Fleck corneal dystrophy (CFD, OMIM 121850) is a rare autosomal dominant stromal dystrophy beginning early in life and is characterized by flat, gray-white, tiny flecks scattered throughout the stroma. The flecks have comma, oval, circular or stellate shapes. Histologically only abnormal keratocytes are involved, which contain excess glycosaminoglycan and complex lipids. Typically, the stroma in between the flecks is clear, and the endothelium, epithelium, Bowman layer, and Descemet membrane are normal. The flecks in CFD may appear as early as at two years of age or even at birth, and generally they are reported not to progress significantly throughout life. Although CFD is thought to be rare, specific incidence figures are difficult to obtain. François and Neetens described the condition for the first time in 1957 [1]. Several reports have proven the autosomal dominant pattern of inheritance of this corneal dystrophy [2-4]. CFD has been linked to chromosome 2q35 [5] due to mutations in the phosphoinositide kinase, five finger-containing (*PIKFYVE*) gene, also known as *PIP5K3* (phosphatidylinositol-3-phosphate/phosphatidylinositol 5-kinase type III) [4]. *PIKFYVE* belongs to a large family of lipid kinases that alter the phosphorylation status of intracellular phosphatidylinositol [6]. The content of phosphatidylinositol 3,5-bisphosphate (PtdIns(3,5)P2) in endosomal membranes changes dynamically with fission and fusion events that generate or absorb intracellular transport vesicles. PIKFYVE is the PtdIns(3,5)P2-producing component of a trimolecular complex that tightly regulates the level of PtdIns(3,5)P2. Other components of this complex are the PIKFYVE activator VAC14 and the PtdIns(3,5)P2 phosphatase FIG4 [7]. In addition to its phosphoinositide 5-kinase activity, PIKFYVE also has protein kinase activity [8]. *PIKFYVE* contains 41 coding exons spanning more than 89 kb, and encodes a 2,089-amino acid protein [4].

In this study we present on the clinical and molecular genetic examinations performed on CFD patients and unaffected relatives in a five generation family of Greek origin.

## Methods

### Family participants

**Figure 1:**
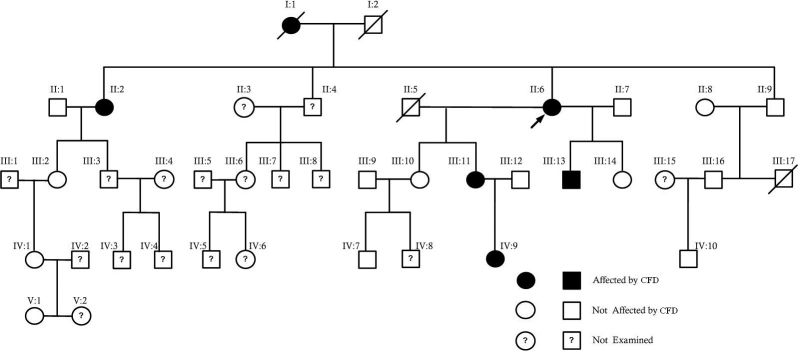
Pedigree of the family with CFD.

A 58-year-old woman (Figure 1, II:6) was seen on routine ophthalmic examination and diagnosed as having CFD. All available family members were examined to evaluate clinical findings and the genetic inheritance pattern of the disease. Twenty members of the family in five generations (Figure 1, Ι:1, II:1, II:2, ΙΙ:6, II:7, II:8, II:9, III:2, III:9, III:10, III:11, III:12, III:13, III:14, III:16, IV:1, IV:7, IV:9, IV:10, V:1) underwent slit-lamp examination. Eleven were females (Figure 1, Ι:1, II:2, ΙΙ:6, II:8, III:2, III:10, III:11, III:14, IV:1, IV:9, V:1) and nine males (Figure 1, II:1, II:7, II:9, III:9, III:12, III:13, III:16, IV:7, IV:10), aged from two years to 85 years old.

### Sequencing and DHPLC analyses

Blood samples were available from four patients with CFD (Figure 1, II:2, II:6, III:11, and III:13) and seven unaffected relatives (Figure 1, II:1, II:7, II:9, III:2, III:10, III:12, and III:14). DNA was isolated from EDTA-anticoagulated blood samples by a standard salting out procedure [9]. Coding exons and adjacent intronic sequences of *PIKFYVE* were amplified from genomic DNA of the proband (Figure 1, II:6) by use of the primers shown in Appendix 1. PCR amplification of each of the 41 exons was performed in 20 μl reactions involving 2.5 U of AmpliTaq Gold (Applied Biosystems, Foster City, CA), 2 μl of 10× AmpliTaq Gold Buffer, 1.9 mM MgCl_2_, 0.25 mM dNTPs, 0.25 mM of primers, and 40 mg of genomic DNA. PCR cycling conditions consisted of an initial 10 min denaturation step at 95 °C for 5 min; 32 cycles at 94 °C for 40 s, 55 °C for 30 s (61 °C for exon 19b), and 72 °C for 40 s; and a final elongation step at 72 °C for 7 min. The PCR templates were sequenced using ABI Dye Terminator (Applied Biosystems, Foster City, CA), version 1, in a final reaction volume of 10 μl. Products of the sequencing reactions were purified using the Performa DTR gel-filtration system (Edge BioSystems, Gaithersburg, MD) and were run on a 3100 ABI genetic analyzer (Applied Biosystems). Sequences were aligned with the reference genomic sequence (GenBank NM_015040) using the Seqman program of the DNASTAR package (DNASTAR, Inc., Madison, WI) or Chromas, version 2.23 (Technelysium Pty Ltd, Brisbane, Australia). The primer sets and annealing temperatures used are shown in Appendix 1. Denaturing high performance liquid chromatography (DHPLC) was used to test co-segregation by verifying the results of direct sequencing of the proband to the other affected patients (Figure 1, II:2, III:11, and III:13). PCR products were screened on a WAVE system (Transgenomics, UK) at a temperature of 60.1 °C and starting at 58.5% B buffer.

Written informed consent was obtained from all participants. The clinical and molecular examinations were in accordance with the Helsinki Declaration.

## Results

**Figure 2:**
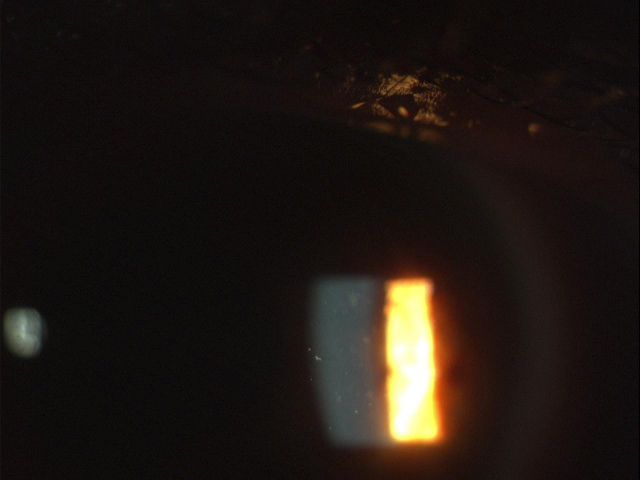
Photo of CFD in case II:6.

**Figure 3:**
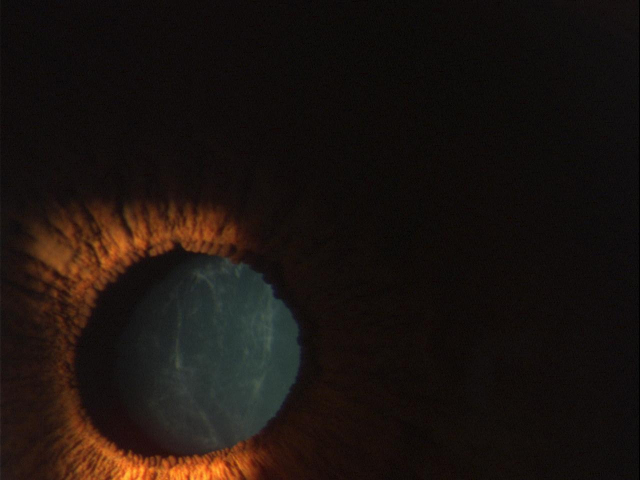
Cataract formation in case II:6.

**Figure 4:**
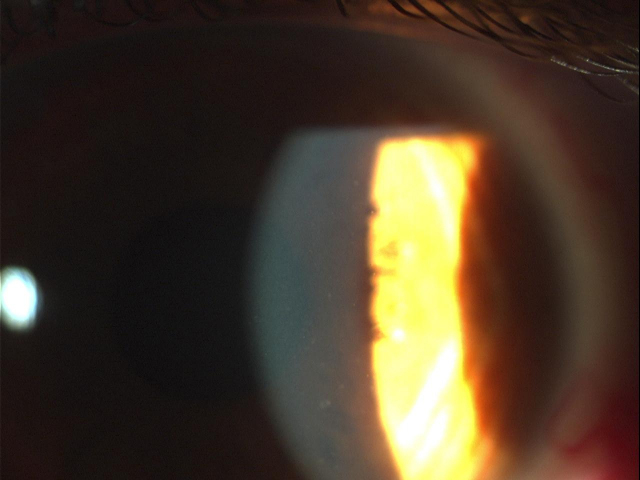
Photo of CFD in case III:11.

**Figure 5:**
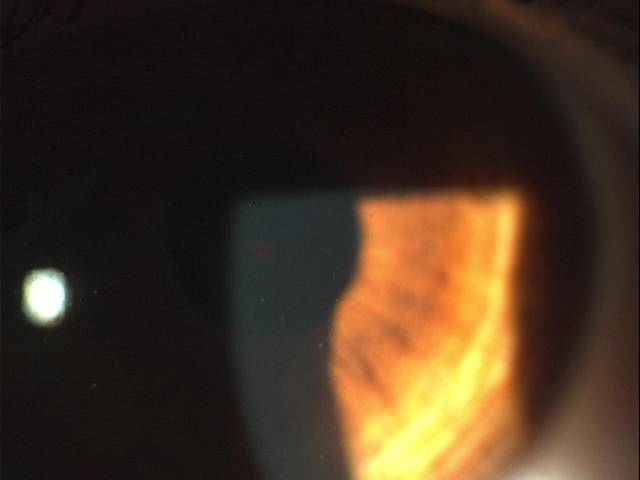
Photo of CFD in case IV:9.

Slit-lamp examination revealed six members of the family affected by CFD (Figure 1, I:1, II:2, II:6, III:11, III:13, and IV:9). In two CFD patients early cataract formation was noticed (Figure 1, II:2 66 years old, and II:6 58 years old). All patients affected by the dystrophy were asymptomatic. Inheritance of the condition in the family was compatible with autosomal dominant inheritance. The clinical signs of patients II:6, III:11, and IV:9 are presented in Figure 2 and Figure 3 (II:6), Figure 4 (III:11), and Figure 5 (IV:9).

**Figure 6:**
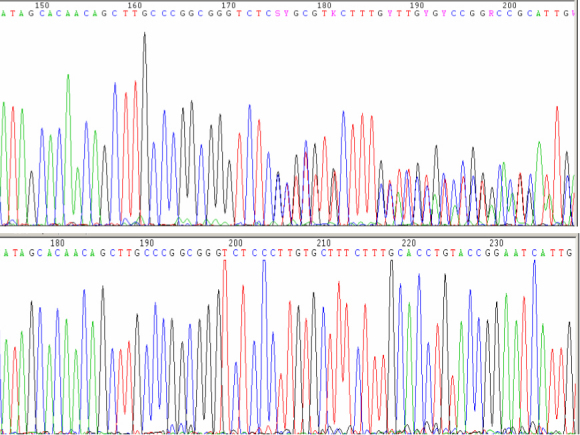
Partial sequence chromatograms of the *PIKFYVE* gene presenting the c.3060–3063delCCTT mutation in heterozygosity (upper panel), and a normal sequence (lower panel).

**Figure 7:**
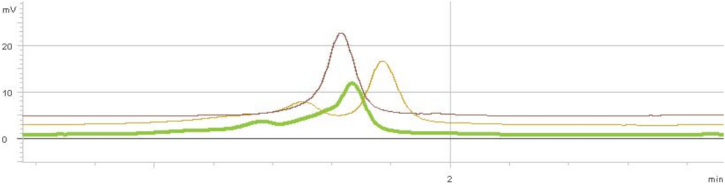
WAVE analysis results. Line with single peak (brown): normal DNA. Line with two peaks (olive): heterozygous c.3060–3063delCCTT carrier (II:2, II:6, III:11, III:13). Line with two peaks (green): DNA from patient II:9 with heterozygous rs2363468 (T>C) SNP and absence of the c.3060–3063delCCTT mutation.

Direct sequencing of the proband (II:6) revealed the existence of a novel heterozygous c.3060–3063delCCTT (p.P968Vfs23) mutation which is a 4-bp deletion in exon 20 of *PIKFYVE* (Figure 6). Co-segregation of the mutation tested by DHPLC using the WAVE technology showed that the c.3060–3063delCCTT (p.P968Vfs23) mutation was present in all family members who were found to exhibit clinical signs of CFD (Figure 1, II:2, III:11, III:13). WAVE results are presented in Figure 7. Normal sequences were detected in unaffected family members II:1, II:9, III:2, III:10, III:12, and III:14 (Figure 1). No molecular screening was performed in the seventh unaffected member (Figure 1, II:7) as the causative mutation was clearly transmitted from his affected wife (Figure 1, II:6) to his affected son (Figure 1, III:13). However, an abnormal sequence was detected by WAVE in the unaffected member II:9 (Figure 7), which subsequently revealed the existence of the non-pathogenic heterozygous rs2363468 (T>C) single nucleotide polymorphism (SNP) by direct sequencing. The c.3060–3063delCCTT (p.P968Vfs23) mutation was not observed in 96 control individuals or in more than 25 patients with CFD previously analyzed in our laboratory.

## Discussion

Mutations in *PIKFYVE* have been implicated as the major cause of CFD, an autosomal dominant human disease characterized by the presence of numerous white flecks scattered in all layers of the stroma [4]. PIKFYVE is a protein important for post-Golgi vesicle processing. Mammalian PIKFYVE is a member of an evolutionarily ancient gene family of PtdIns(3,5)P2-synthesizing enzymes that are large proteins, represented by a single-copy gene in most, if not all, species with sequenced genomes, including the zebrafish [10,11]. Most of the reported mutations are of nonsense and frameshift type and occur in and around the Cpn60_TCP1 domain, predicting termination of the peptide before the catalytic domain. Although the activity of the PIKFYVE protein or the PtdIns(3,5P)2 levels have not been determined under these conditions, it is expected that the mutations will result in a loss of PIKFYVE function. Given the lethality of the *PIKfyve* null mutations in model organisms [10], the asymptomatic condition of the CFD patients is quite surprising but could be attributed to the presence of one normal allele. Histological findings in CFD indicating that the corneal flecks are swollen keratocytes containing membrane-limited empty vacuoles [12], are consistent with the phenotypic changes seen under perturbed PIKfyve functionality [10].

In this study we reported our findings on the clinical evaluation and the molecular genetic examination of a family presenting CFD. To our knowledge, this is the first reported family of Greek origin. The pedigree (Figure 1) consisted of a total of 40 individuals in five generations, among which 20 were subjected to clinical evaluation, whereas 20 were not. Among the 20 examined, six were found to be affected by CFD (Figure 1, I:1, II:2, II:6, III:11, III:13, and IV:9), and 14 were not affected (Figure 1, II:1, II:7, II:8, II:9, III:2, III:9, III:10, III:12, III:14, III:16, IV:1, IV:7, IV:10, V:1). Early cataract was noticed in two CFD patients (Figure 1, II:2 and II:6). Four individuals are deceased, including one affected (Figure 1, I:1) and three not examined persons (Figure 1, I:2, II:5, III:17). Mutational screening of the *PIKFYVE* gene by direct sequencing in the proband (Figure 1, II:6) revealed the existence of the novel mutation c.3060–3063delCCTT in heterozygosity (Figure 6). This 4-bp deletion was further found to be the cause of a frameshift leading to the p.P968Vfs23 alteration on the protein level. The mutation was confirmed in all affected patients from which bloods were drawn (Figure 1, II:2, III:11, III:13) by DHPLC analysis using the WAVE technology. We conclude that the novel c.3060–3063delCCTT (p.P968Vfs23) mutation in *PIKFYVE*, which segregated with the disease, was the causative mutation in this family.

## Appendix 1. PCR Primers for 41 exons of *PIKFYVE* (primers for DHPLC).

To access the data, click or select the words “Appendix 1.” This will initiate the download of a compressed (pdf) archive that contains the file.
